# A Rare Case of Primary Clear Cell Adenocarcinoma of the Urinary Bladder

**DOI:** 10.7759/cureus.39575

**Published:** 2023-05-27

**Authors:** Areen Amleh, Roaa Mubarak, Hadeell Jameel Ayesh, Layth Al-Karaja, Mohammed Ayyad, Mahmoud Adnan Allan, Motaz Natsheh

**Affiliations:** 1 Surgery, Al-Quds University, Jerusalem, PSE; 2 Internal Medicine, Al-Quds University, Jerusalem, PSE; 3 Urology, Princess Alia Hospital, Hebron, PSE; 4 Pathology, National Pathology Laboratory, Hebron, PSE

**Keywords:** urology, hematuria, cystoscopy, bladder cancer, adenocarcinoma

## Abstract

Adenocarcinoma is a rare form of urinary bladder cancer, comprising only 2% of cases, with various histological patterns and levels of differentiation. Among these, clear cell adenocarcinoma is the least common. Contrary to other subtypes, clear cell adenocarcinoma of the bladder has been shown to have a female predominance, and typically presents at the age of 60 after being incidentally discovered on radiological and urinary studies. However, signs and symptoms such as visible and non-visible hematuria, and signs and symptoms of urinary tract infection refractory to antibiotic treatment could occur and clue into the diagnosis. Although imaging can reveal and characterise the lesion, definitive diagnosis requires cystoscopy with biopsy. The treatment of adenocarcinoma of the bladder often requires surgical resection, with adjuvant chemotherapy being utilized in a subset of patients.

We report a 79-year-old patient complaining of gross hematuria. Ultrasound was performed and showed a calcified mass at the dome of the urinary bladder, which was confirmed by computerized tomography of the abdomen and pelvis. Subsequent cystoscopy confirmed the diagnosis of clear-cell adenocarcinoma and the tumor was resected using a trans-urethral approach. Radical cystectomy with regional lymphadenectomy and adjuvant chemotherapy were used as the primary therapeutic modality.

## Introduction

Primary clear cell adenocarcinoma (CCA) of the bladder is an extremely rare form of bladder cancer, accounting for less than 1% of cases [1]. Its aggressive nature and limited understanding due to its rarity have contributed to a lack of comprehensive knowledge regarding its clinical behavior and optimal treatment strategies. Urothelial carcinomas are the most common type of bladder tumors, while adenocarcinomas and squamous cell carcinomas are less frequently encountered [2]. Given its distinct clinical, pathological, and therapeutic features, CCA holds significant importance as a subject of investigation in the field of urologic oncology [1,2].

We herein present a case of a 79-year-old female patient who was diagnosed with primary clear cell adenocarcinoma of the urinary bladder. The case description includes information about the patient's symptoms, diagnostic evaluation, and the treatment approach adopted for this uncommon tumor. Additionally, we provide a comprehensive review of the existing literature on primary clear cell adenocarcinoma of the urinary bladder, encompassing its epidemiology, histological characteristics, and currently used treatment modalities.

## Case presentation

**Figure 1:**
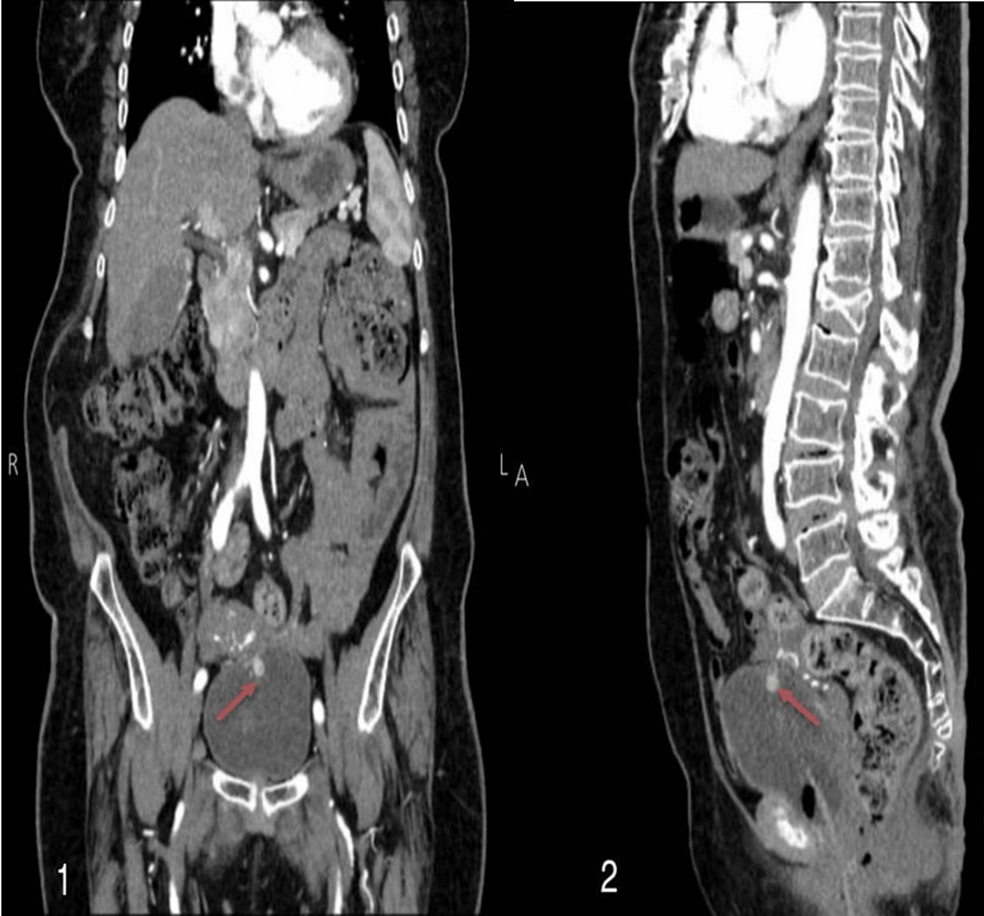
CT scan of the urinary tract showing a polypoid lesion protruding from the dome of the urinary bladder. CT scan of the urinary tract showing a vividly enhanced polypoid lesion measuring about 1 cm protruding from the dome of the urinary bladder. (1) Arterial phase - coronal section; (2) Delayed phase - sagittal section.

A 79-year-old female presented to the outpatient clinic complaining of painless visible hematuria of unknown duration. She denied experiencing any other symptoms such as urgency, frequency, dysuria, or constitutional symptoms. Her medical history included diabetes mellitus, hypertension, and ischemic heart disease. Physical examination was unremarkable. Laboratory tests revealed a hemoglobin level of 11.5 g/dL (12.1 to 15.1 g/dL), with a normal metabolic panel and creatinine level. Urine analysis showed no significant abnormalities except for the presence of numerous red blood cells. Radiological imaging, including a pelvic ultrasound, detected a polyp-like mass measuring around 1.4x1.4 cm on the right side of the urinary bladder's dome, which exhibited punctate calcifications and increased vascularity. Subsequently, a contrast-enhanced computed tomography (CT) scan was performed, revealing a 1 cm prominently enhanced polypoid lesion protruding from the bladder's dome (Figure 1). Another non-enhancing lesion was observed within the bladder, containing a dense spot, indicative of a urinary bladder clot. Cystoscopy confirmed the presence of a smooth-surfaced papillary mass located in the dome of the bladder. A transurethral resection of the tumor with biopsy was performed for further investigation.

**Figure 2:**
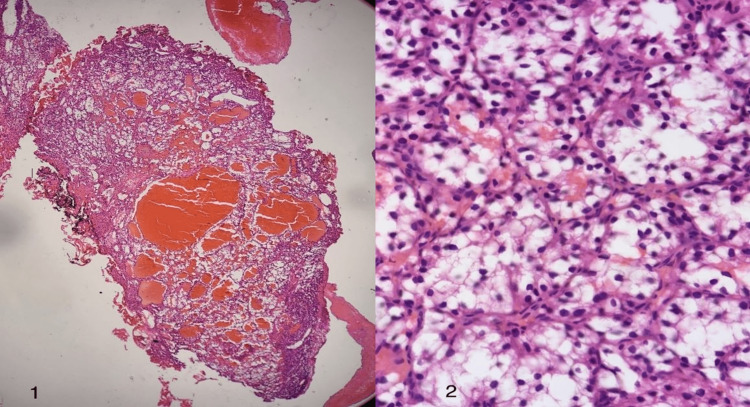
Microscopic view of the resected bladder neoplasm. Hematoxylin and Eosin stain; Microscopic examination shows that the tumor is composed of tubulocystic structures lined by cuboidal cells with abundant clear cytoplasm. (1) Low power microscopic view; (2) High power microscopic view.

**Figure 3:**
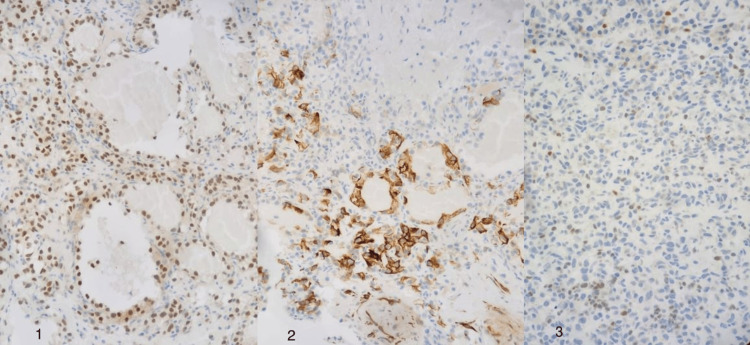
Immunohistochemical staining of neoplastic cells. The neoplastic cells displayed positivity for PAX8 and CK7 immunostains, as well as the presence of scattered immunoreactive cells with GATA3 immunostain. (1) PAX3 immunostain; (2) CK7 immunostain; (3) GATA3 immunostain.

Postoperatively, the resected tissue specimen was histologically examined and showed a highly vascular tumor composed of cuboidal cells arranged in a tubulocystic pattern with large hyperchromatic nuclei (Figure 2). The neoplastic cells were positive for PAX8 and CK7 (focal) immunostains (Figure 3). GATA3 immunostain highlighted scattered immunoreactive cells, while P63 and CK20 immunostains were negative. These findings supported the diagnosis of bladder clear-cell adenocarcinoma.

Further management and staging of the patient’s tumor was performed. The stage of the tumor according to the TNM system was T2N1M0. Partial cystectomy was offered, however, the patient refused the procedure due to the strict follow-up needed after surgery. In light of her age and medical history, radical cystectomy with regional lymphadenectomy was performed. The urinary diversion method used was a cutaneous uretero-ureterostomy. Postoperative histopathology report revealed two positive lymph nodes. Subsequently, the patient was started on cisplatin-based chemotherapy. The patient was followed up six months and one year postoperatively with no clinical or radiological evidence of tumor recurrence.

## Discussion

Primary clear cell adenocarcinoma of the urinary bladder is a rare tumor, with fewer than 50 cases documented in the literature [3]. Of all the subtypes, transitional cell carcinoma is the most common variant of bladder neoplasms, accounting for 90% of cases, while squamous cell carcinoma and adenocarcinoma together make up approximately 9% of cases [4]. Adenocarcinoma has multiple subtypes based on the histological appearance, including clear cell, enteric, adenocarcinoma not otherwise specified (NOS), signet ring cell, mucinous, hepatoid, and mixed subtypes [5]. Furthermore, clear cell adenocarcinoma has a female-to-male ratio of 3:2, with a mean age of presentation of around 60 years (range 19-83 years) [6]. The most common presenting sites are the neck of the bladder followed by the posterior wall [7]. Signs and symptoms of this neoplasm are usually nonspecific, and can include microscopic or macroscopic hematuria, as well as symptoms of recurrent urinary tract infections [7]. Although radiological evidence can aid in diagnosis, cystoscopy is often necessary to confirm and characterize malignant lesions of the urinary bladder. Clear-cell adenocarcinoma can manifest as either papillary or sessile lesions [8]. Its clinical presentation is usually nonspecific, highlighting the importance of relying on microscopic evaluation for diagnosing the adenocarcinoma type. 

Several hypotheses have been raised regarding the histogenesis of clear-cell adenocarcinoma. The older theory suggests that it is of mesonephric origin, but this theory has been rejected due to lack of convincing evidence. Other theories suggest that clear-cell adenocarcinoma is of Müllerian origin, as neoplasms have occasionally been linked to vesical endometriosis or have developed in cysts or remnants of the Müllerian duct in the bladder [9]. Authors theorize that it developed from Müllerian elements in the bladder and is histogenetically identical to the female genital tract. This theory was accepted for a long time until a recent study revealed that the majority of clear cell carcinomas have displayed evidence of urothelial origin [10].

Microscopically, clear-cell adenocarcinoma appears as a mixture of tubular glands, papillae, microcysts, and diffuse masses. The cells vary in shape from flat to hobnail to cuboidal and have a large amount of transparent, glycogen-rich cytoplasm [11]. They frequently exhibit substantial nuclear atypia [11]. Immunohistochemical staining can be helpful in differentiating between the various subtypes; as clear cell adenocarcinomas typically stain positive for CK7, while CK20 expression is more variable [6]. It's critical to distinguish clear cell adenocarcinoma from other malignant tumors, such as urothelial carcinoma with clear cells and metastasis from the ovary, both of which stain positive for CK7 and CK20, as well as differentiating it from renal metastasis, in which both stains are negative.

## Conclusions

Primary clear cell adenocarcinoma of the urinary bladder is an extremely rare, aggressive, and unfavorable neoplasm with a poor prognosis. The tumor often presents at an advanced stage, making it challenging to treat with high likelihood of disease progression, metastasis, and recurrence. Treatment of clear cell adenocarcinoma of the urinary bladder often involves a multimodal approach with surgical resection being the primary modality implemented. This case demonstrates the significance of including clear cell adenocarcinoma in the differential of bladder malignancies and showcases the importance of histopathological evaluation in guiding further management.
